# Recent advances in the detection and functional analysis of circRNAs with short-read RNA sequencing-based methods

**DOI:** 10.1007/s11033-026-12267-y

**Published:** 2026-07-03

**Authors:** Grace Lindner, Si-Mei Xu, Kristina Santucci, Yulan Gao, Michael Janitz

**Affiliations:** School of Biotechnology and Biomolecular Sciences, University of New South Wales, Sydney, NSW 2052 Australia

**Keywords:** Circular RNAs, Protein-coding potential, Backsplicing, Bioinformatics tools, Machine learning

## Abstract

Circular RNAs (circRNAs) were first identified approximately 50 years ago in pathogenic viroids as single-stranded, covalently closed RNA molecules. Initially considered by-products of splicing, circRNAs are now recognised as an important class of regulatory RNAs involved in microRNA sponging, RNA–protein interactions, and cellular pathways. Their closed-loop structure, generated through backsplicing, confers resistance to exonucleolytic degradation and contributes to their stability. Owing to their tissue- and disease-specific expression, circRNAs have emerged as promising biomarkers for cancer, neurodegenerative disorders, and cardiovascular disease. Over the past decade, numerous bioinformatics tools utilising RNA-sequencing (RNA-seq) data have been developed for circRNA detection and analysis. Detection methods have evolved from manual split-read inspection to automated identification of the back spliced junction, while annotation pipelines now resolve the genomic origins and structural characteristics of circRNAs. Because individual circRNA callers vary considerably in sensitivity and specificity, a combined usage of tools in circRNA detection has become the preferred strategy for generating high-confidence datasets. Beyond their non-coding functions, increasing evidence suggests that some circRNAs possess protein-coding potential through open reading frames, cap-independent translation mechanisms, internal ribosome entry sites (IRESs), and N6-methyladenosine modifications. A new generation of bioinformatic tools can now assess the protein-coding potential of circRNAs, integrating the above features, as well as machine learning and deep learning approaches refining these predictions. This review summarises recently developed short-read RNA-seq bioinformatics tools for circRNA detection, consensus calling, annotation, and protein-coding potential prediction, with a particular focus on advances from the past five years that facilitate the identification of translatable circRNAs.

## Introduction

The human genome is predominantly composed of non-coding RNAs (ncRNAs), despite the greater prevalence of studies focusing on the biological implications of protein-coding genes. Circular RNAs (circRNAs) are single-stranded RNA molecules formed by backsplicing, in which the 5’ and 3’ ends of linear RNA are linked, resulting in the formation of a circular structure. Their unique circular structure makes them remarkably stable and resilient to exonucleolytic degradation [1]. To date, researchers have identified thousands of circRNAs in eukaryotic transcriptomes, indicating their involvement in complex and regulatory functions in numerous cellular processes.

The biogenesis of circRNAs is regulated by splicing factors or intronic complementary sequences, indicating that their expression is not merely a by-product of splicing events but also a direct outcome of cellular signalling [2]. It is a multifaceted process involving various mechanisms, primarily characterised by backsplicing and lariat formation. Specific *cis*-acting elements are involved in circRNA biogenesis, in which regions of non-coding DNA regulate the transcription of neighbouring genes and trans-acting factors. A *trans*-acting factor binds to a *cis*-regulatory element that causes changes in transcriptional expression levels. Furthermore, Recent studies have also shown that their biogenesis requires spliceosomal machinery modulated by the *cis*-acting elements and protein factors. CircRNAs are endogenous biomolecules with tissue-specific and cell-specific expression patterns. Many circRNAs are evolutionarily conserved and can act as microRNA (miRNA) or protein inhibitors (‘sponges’) by regulating protein function or by being translated.

While most circRNAs are non-coding and are involved only in regulatory mechanisms, a subset of circRNAs is considered translatable [3]. Recent technological advancements have enhanced the detection and functional analysis of circRNAs and their protein-coding potential. Initiated by internal ribosome entry sites (IRESs) and N6-methyladenosine modifications (m^6^a), circRNAs with opening reading frames (ORFs) can undergo cap-independent translation. The protein-coding potential can be calculated using specific algorithms, such as logistic regression, and specific features that analyse whether a given ORF can be translated. The stability of circRNAs in tissues suggests that they could be promising candidates for non-invasive biomarkers, offering new perspectives on disease detection and monitoring.

Elevated or altered levels of specific circRNAs have been associated with several diseases, including various cancers, neurodegenerative disorders and cardiovascular diseases, such as glioma, gastric, T-cell acute lymphoblastic leukaemia, hepatocellular carcinoma, breast cancer, lung cancer, bladder cancer, oesophageal, prostate, colorectal and bowel cancer [4].

circSHPRH has been found to affect glioma cancers by overexpressing and reducing their malignant behaviour such as tumour cell proliferation, migration and invasion [5]. circLINC-PINT has been associated with glioblastoma by suppressing proliferation in vitro and in vivo [6]. CircRNA circβ-catenin has been noted to promote liver cancer cell growth by activating the Wnt pathway [7]. It has been found abundantly in liver cancer in contrast to its healthy tissue counterpart. CircFBXW7 has been associated with breast cancer and glioma, in which the knockdown of the transcript increases malignant phenotypes [8]. Upregulation of CircPPP1R12A in colon cancer cells has been known to affect the migration, proliferation and invasion rendered by the Hippo-YAP signalling pathway [9]. CircFNDC3B has been identified in various carcinomas, such as colon, oesophageal and bowel tissues [10–12]. It has been found to promote the angiogenic activity of oral squamous cell carcinoma and inhibit the proliferation, invasion and migration of colon cancer cells. In Ghafouri-Fard et al.’s 2021 study, circFGFR1 was upregulated in lung cancer samples. CircRNAs act as sponges for miR-381-3p to regulate the expression of the C-X-C motif chemokine receptor 4, a protein ontributes to the progression of non-small cell lung carcinoma and resistance to targeted immunotherapies [13].

The human brain is highly enriched in circRNAs, with dynamic changes in expression eluding to neural activities [14]. Consequently, circRNAs have been detected in neurodegenerative diseases such as Alzheimer’s disease (AD) and Parkinson’s disease (PD). Song et al.’s study in 2022 discovered circCwc27 as a novel circRNA involved in AD found to directly bind purine-rich element-binding protein A (Pur-α), which suppresses Pur-α recruitment, to the promoters of AD-associated genes including the amyloid precursor protein [15]. Another study found 70 dysregulated circRNAs in AD patients, with 11 circRNAs forming relationships with circRNA-miRNA-mRNA regulatory networks [16]. For PD, a study in 2022 found an association with circSV2b participating in oxidative stress that regulates the miR-5107-5p-Foxk1-Akt1 axis [17]. circSV3b directly sponged miR-5107-5p and alleviated the suppression of the expression of Foxk1, which positively regulated Akt1 transcription. Another study identified a higher expression of hsa_circ_0004381 expression in PD patients when compared with control samples [18]. Chen et al. analysed circTLK1 by investigating the miR-26a-5p network and found a higher expression in the brain tissue of a PD mouse model compared with the control [19].

In recent years, circRNAs have also been investigated in cardiovascular diseases, such as atherosclerosis, myocardial infarction, coronary heart disease, cardiac senescence and hypertensive renal injuries [20, 21]. Together, these studies underscore the growing recognition of circRNAs as key regulators in cancer, neurodegeneration and cardiovascular pathology, with increasing evidence supporting their potential clinical utility. The detection and downstream functional analysis of circRNAs now depends on the development of computational methods which utilise RNA-seq reads. Particularly, the translation of circRNAs and determining their protein-coding potential has drawn interest.

This review focuses specifically on bioinformatic tools that span the circRNA detection-to-translation analysis pipeline, covering the foundational detection algorithms and the most recent generation of coding-potential predictors. In contrast to prior benchmarking studies that have focused on circRNA detection alone [22], the present study integrates the evaluation of detection, annotation and coding potential prediction tools whose outputs are compatible across pipeline stages and support the interpretation of translatable circRNA candidates. The tools described in this review are organised according to their role in the circRNA analytical pipeline. Single-tool detection algorithms that identify back spliced junctions (BSJs) from RNA sequencing (RNA-seq) data are presented first and then grouped according to their underlying alignment strategy. Meta-pipeline frameworks that integrate multiple callers to improve robustness are then discussed, followed by recent cross-tool benchmarking studies that inform tool selection at the detection stage. Tools used to annotate and visualise detected circRNAs are subsequently examined, and the prediction of protein-coding potential as the final analytical layer is portrayed. This pipeline-based organisation reflects the order in which tools are typically applied in a circRNA analysis workflow and allows for a direct comparison of methods at each analytical stage.

## Tools for circRNA detection and quantification

The discovery of circRNAs in humans was first documented in 1991, in which an RNA expression assay revealed that a transcript derived from a gene deleted in colon cancer was produced from noncanonical splicing [23]. The number of studies on circRNA discovery has increased with the development of RNA-seq technology. Over the past two decades, the upsurge in publicly available short-read RNA-seq data has allowed for mass circRNA detection and analyses [24]. Modern bioinformatics aims to overcome the challenge of translating a large quantity of sequencing data into comprehensible biological knowledge.

### The development of circRNA detection pipelines using short-read sequencing approaches

**Fig. 1:**
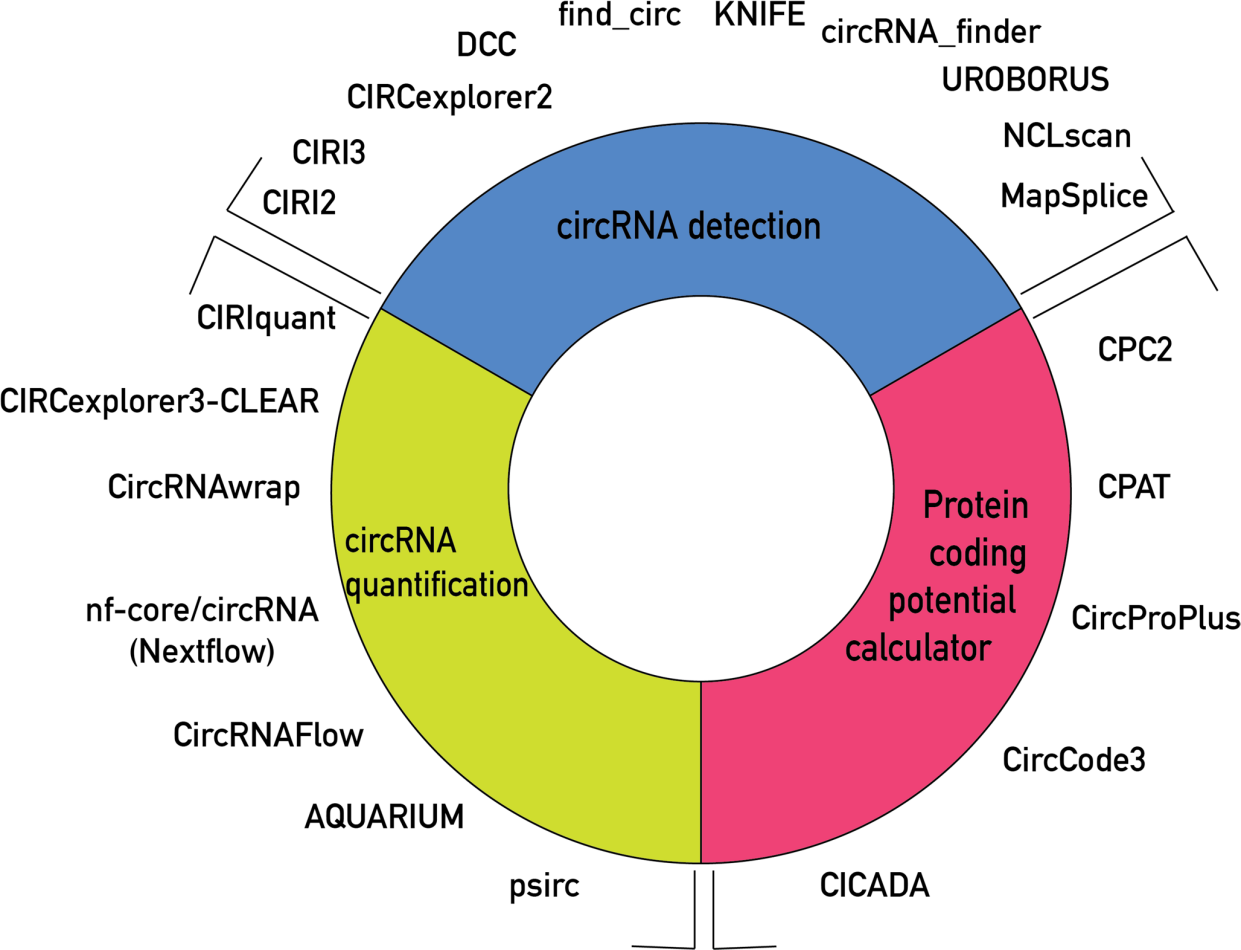
Primary functions of the bioinformatics tools used to analyse circRNAs. The programmes and pipelines shown in the figure are categorised into three types: circRNA detection, circRNA quantification and calculation of the protein-coding potential. Figure created by Adobe Illustrator

**Fig. 2:**
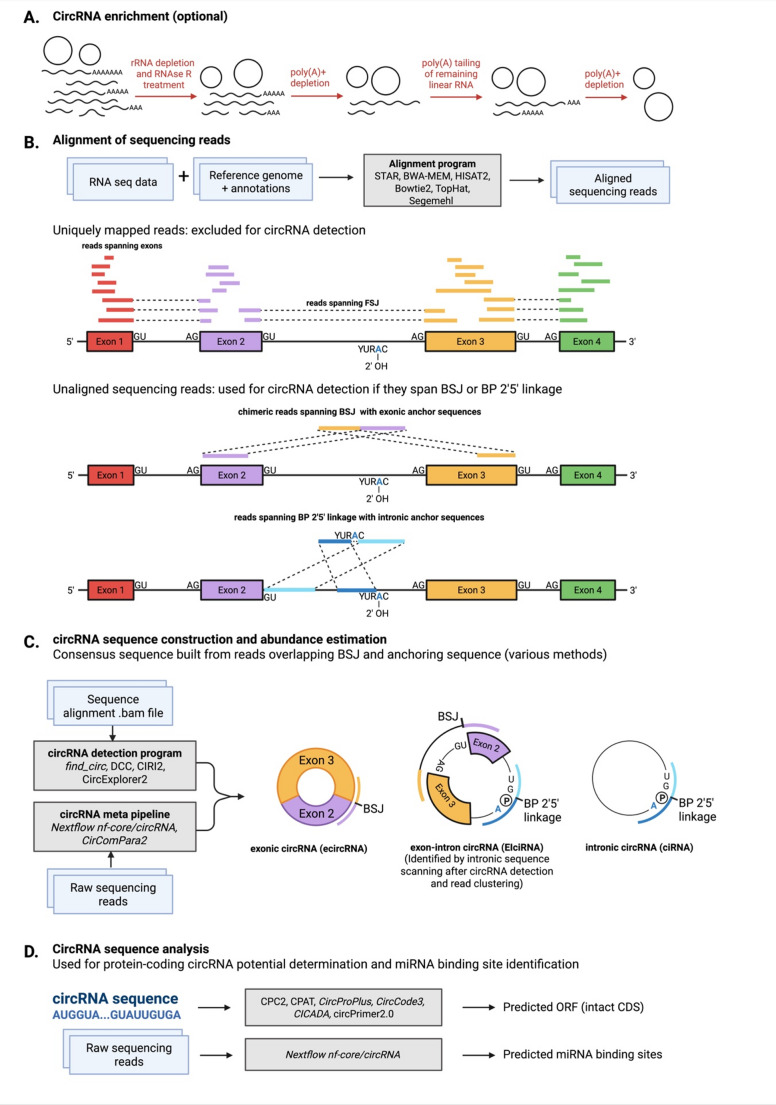
Bioinformatic detection of circRNAs following Ribo-seq or RNA-seq. (**A**) The RNA-seq of total RNA or the RNA-seq of circRNAs enriched in specific library preparations can be used for circRNA detection through downstream bioinformatic analyses. (**B**) This analysis is facilitated by circRNA detection programmes that scan reads that are not uniquely mapped to or have not been aligned with a reference genome. CircRNAs can be detected by inspecting such reads and determining whether they contain features such as BSJs or branch points (BPs) (given that the anchoring sequences are in reverse order to those of the reference genome). (**C**) The circRNA sequence is obtained by extending such reads and anchoring sequences, which is performed using a variety of programmes with different underlying algorithms (described in the main text; see Sects. 2–5). (**D**) The inspection of such circRNA sequences using protein-coding prediction tools (described in the main text; see Sects. 6–7) allows the determination of protein-coding circRNAs and the prediction of the ORF. Similarly, the sequence can be scanned for miRNA binding sites. Figure created by BioRender.com.

With advancements in bioinformatics approaches over the past 10 years, new computational methods have been developed to detect, quantify and assess the protein-coding potential of circRNAs in RNA-seq data (Fig. 1). Earlier circRNA detection methods, such as northern blotting, targeted PCR approaches, RNase resistance assays and cloning, relied on the manual inspection of BSJ reads, which was labour intensive and prone to a high occurrence of false positives [25]. Novel assays for circRNAs, such as droplet digital PCR, isothermal exponential amplification and rolling cycle amplification, have also recently emerged. Today, specialised computational pipelines, such as CIRI [26], find_circ [27], CIRCexplorer2 [28] and CIRIquant [29], automate the detection of circRNAs by leveraging split-read mapping strategies that detect BSJs from RNA-seq and, less commonly, ribosome sequencing (Ribo-seq) data (Fig. 2). Ribo-seq data are used to calculate protein-coding potential as a segment arising from a splice junction, and from the BSJ, it can be analysed for translation [30]. By aligning Ribo-seq data to identified circRNA sequences, ribosome-specific fragment mapping to BSJ regions can be performed, providing evidence of the active translation of the circRNA. These methods incorporate various filters to exclude sequencing artefacts and repetitive regions to improve the sensitivity and specificity of circRNA detection (Table 1). Using a combination of experimental techniques and bioinformatics approaches, the validation of circRNAs can be accomplished (Fig. 1).

Vromman et al.’s benchmark study extensively described 16 circRNA detection tools and reported that multiple tools can be integrated to complement each other, increasing detection sensitivity and accuracy [22]. The study also established a list of recommendations for circRNA detection and validation. In comparison, this review evaluates recently developed tools for circRNA detection, quantification and prediction of the protein-coding potential, with a focus on their methodological approaches and applicability to identifying translatable circRNAs.

**Table 1:** List of circRNA detection tools

Tool	Aligner	Methods used to identify BSJs	Language	Studies applying the tool
CIRI2	BWA-MEM	Multiseed matching, maximum likelihood estimation-based algorithm	Perl	[22, 31–39]
CIRI3	BWA-MEM or STAR	Maximum likelihood estimation, differential expression with CIRIquant/edgeR, quasi-likelihood ratio test, rMATS algorithms	Java	N/A
CIRCexplorer2	STAR/segemehl/MapSplice/TopHat-Fusion	Parse chimeric/split reads, realignment near splice sites, annotation of BSJs and alternative backsplicing	Python	[32, 34, 36, 37, 40–43]
DCC	STAR	Chimeric junction-based BSJ detection with stringent postfilters	Python	[44–47]
find_circ	Bowtie/Bowtie2	Anchor-based split alignment around BSJs, filters mismatches and repeats	Python	[45, 47–50]
KNIFE	Bowtie/Bowtie2 to junction index	Circular junction index + generalised linear model	Python, R, Perl	[47, 49, 51]
circRNA_finder	STAR	Parse Chimeric.out.junction, filters repeats and low support	Python	[22, 50, 52]
MapSplice	Splice-aware aligner	Splice detection algorithm for noncanonical junctions and other novel splicing events	C++, Python, Perl utilities	[47, 50]
UROBORUS	TopHat and Bowtie	Forms an artificial paired-end seed (20 bp) and aligns with a reference genome	Perl	[53]
NCLscan	BWA, Novoalign and BLAT	Stepwise alignment strategy to detect non-collinear sequences from non-poly(A) selected sequences that present intragenic events	C++, Python	[54–56]

Abbreviations: BSJ – back-splice junction

## Early development of circRNA identification tools

The earliest tools developed for circRNA detection relied on relatively simple read-anchoring or chimeric-alignment strategies. These methods established the algorithmic foundations on which more recent detection tools have been built from.

### Detection of circRNAs through anchor-based back splice mapping

find_circ was one of the earliest tools developed to identify circRNAs by detecting the BSJs from RNA-seq data [27]. The programme first aligns RNA-seq reads using Bowtie, a fast short read aligner and predecessor to Bowtie2 [57]. It identifies unmapped reads suspected to be BSJs and scans them for anchored segments, which are short sequences that can be read and independently aligned to the genome in reverse order. A series of filtering steps are then used to reduce false positives, such as ensuring that more than one sequencing read supports the BSJ and that the breakpoint within the junction is unambiguous. The output provides the genomic coordinates of the circular junction, the number of supporting reads and other alignment features. Although the programme is efficient, it does not account for strand specificity or detailed exon structures and yields a higher false-positive rate than tools such as CIRI2 and CIRCexplorer2 [26]. Nonetheless, find_circ was the foundation of early circRNA detection tools and remains a lightweight option for an initial circRNA screen.

### Detection of chimeric circRNAs

Detection of chimeric circular RNAs (DCC) is another programme that detects circRNAs from chimeric reads produced by the spliced transcripts alignment to reference (STAR) aligner [58]. The principal feature of the DCC workflow is the utilisation of the chimeric output of the STAR aligner, which contains split reads that map in a nonlinear manner to the genome, a hallmark of backsplicing in circRNAs. Potential circular junctions are indicated by reads with anchor sequences that align in reverse order. DCC applies a series of stringent filters to reduce false positives, such as a mapping quality threshold, excluding junctions near splice sites of linear transcripts and filtering junctions present in low-complexity or repetitive regions. CircRNAs from annotated genes can be detected by providing gene annotation files, such as those from GENCODE, a project that classifies all gene features in human and mouse genomes [59]. Detecting circRNAs from annotated genes is advantageous because their exon composition can be determined and can facilitate the identification of exonic, intronic and intergenic circRNAs. The number of unique BSJ reads is also quantified, supporting circRNA detection. DCC also estimates circRNA versus host gene expression by counting junction and non-junction reads. The read counts are used to test for host gene independence of circRNA expression across different experimental conditions using the R package CircTest. In 2016, Cheng et al. compared the performance of DCC with that of CIRI and KNIFE using several RNA-seq datasets [58]. DCC predicted fewer circRNA candidates than KNIFE did, but the precision was higher, with DCC reaching 97%, followed by a precision of 89% for CIRI and 41% for KNIFE. Moreover, DCC resulted in the greatest number of true positive predictions. Overall, DCC provides a robust statistical framework based on beta-binomial distribution, enabling the detection of host gene-independent changes in circRNA expression.

## Established detection and annotation tools: CIRI2/3 and CIRCexplorer2/3

Building on these early anchor and chimeric read approaches, two predominant tool families have been utilised for circRNA detection: the CIRI series and the CIRCexplorer series. Both rely on more sophisticated alignment strategies and have been continuously updated to improve sensitivity, specificity and analytical depth.

### CIRI2

Circular RNA Identifier version 2 (CIRI2) is one of the gold standard tools used in bioinformatics to detect circRNAs in RNA-seq data with high accuracy and efficiency [26]. CIRI2 is built on the original CIRI algorithm with improved sensitivity and specificity while maintaining computational speed [60]. The main approach for circRNA detection by CIRI2 is the identification of BSJ reads by analysing RNA-seq reads that have been aligned to a reference genome using Burrows–Wheeler aligner-maximum exact matches (BWA-MEM) [61], an aligner capable of accurately mapping chimeric and split reads. CIRI2 works by taking processed single or paired read fastq files generated by RNA-seq, which have been trimmed using Trimmomatic and aligned using BWA-MEM [62]. CIRI2 scans the alignment files for paired chiastic clipping signals (PCCs) and then extracts candidate circRNA junctions based on these split-read alignments. The reads and the presence of canonical splice site signals, such as GU/AG at junction sites, are subsequently filtered. This step is followed by the validation of the circular structure, with the first paired end read crossing the BSJ and the other paired read mapping within the circRNA body. False positives are reduced by excluding circRNAs from repetitive or low-complexity regions. Like DCC, when provided with a gene annotation file, CIRI2 can map circRNAs to known genes and classify them as exonic, intronic or intergenic based on their genomic location.

Greater accuracy in annotation can be accomplished through the determination of the strand of origin for each circRNA. The output file provides the genomic coordinates of the BSJs, the number of supporting reads and the relevant gene annotation information. Gao et al., the developers of CIRI2, benchmarked the performance of their programme against that of CIRI. When CIRI2 was compared with CIRI, 3,041 circRNAs were shared, and CIRI2 was predicted to have 148 more candidates, which were not identified using CIRI. Their predicted BSJ read count was quantified with and without RNase R treatment to determine whether it was a true positive. They found that CIRI2 could effectively remove more false positives in CIRI. However, the true positives remained largely unchanged, indicating the balanced performance of CIRI2 in terms of both false discovery rate (FDR) and sensitivity. The researchers reported that CIRI2 outperformed all the tested programmes in detecting circRNAs and could not be replaced by any of the eight algorithms. Overall, CIRI2 is a robust and annotation-independent method for circRNA detection, allowing researchers to identify novel circular transcripts and quantify their expression.

### CIRI3

Published in 2025, CIRI3 is the updated and more comprehensive version of CIRI2, and it allows for the exploration of differential expression in addition to the quantification of circRNAs [49]. CIRI3 has been optimised for rapid circRNA detection from multisample alignment results, with higher accuracy and improved runtime and memory efficiency. It enables the identification of intronic self-ligated circRNAs and the use of a circRNA list as input for targeted quantification. Unlike CIRI2, CIRI3 uses a conjunction of the BWA and STAR aligner, which increases the capture of reads by identifying BSJs from PCC signals and chimeric splice junctions [63]. The outputs from CIRI3 include BSJ information, forward splice junction (FSJ) reads, differential expression, differential splicing and differential circularisation. The updated workflow of CIRI3 consists of two primary alignment-scanning modules—high-confidence BSJ discovery and FSJ/BSJ read recovery. The first scan examines the PCC clipping signals and filters them by matching splice signals flanking the putative BSJ. Exon and intron boundaries for splicing signal filtrations can also be extracted when a GTF/GFF annotation is provided. The second scan uses a blocking search approach to recover missed BSJ reads and to identify FSJ reads. FSJs read the linear splice junctions, the linear isoform counterpart to the BSJ of the circRNA. For the analysis of differential expression, CIRI3 integrates algorithms from CIRIquant, which applies a generalised fold change approach for datasets lacking biological replicates and a quasi-likelihood ratio test using edgeR with the trimmed mean of M-value normalisation for replicated datasets. Differential splicing and circularisation analyses are performed through the integration of rMATs [64].

The developers of CIRI3 compared their version with five commonly used circRNA tools: find_circ, KNIFE, CIRCexplorer3, DCC and CIRI2 [26, 27, 58, 65, 66]. Overall, they found that CIRI3 had a higher putative positive rate and a lower false-positive rate [49]. When CIRI3 was compared with CIRI2, a significant overlap of 4,337 circRNAs was detected, with 109 outliers identified using CIRI3 and 17 identified using CIRI2. This result may be due to the integration of the Smith‒Waterman alignment, which recovers BSJ reads missed by CIRI2. CIRI3 implements optimised memory management to prevent resource bloat by loading only essential alignment data into memory at any given time instead of keeping the entire dataset resident, as earlier tools often did. Combined with the block-based structure, the memory usage of CIRI3 increases more slowly with the dataset size compared with other tools. Another critical improvement is the blocking search strategy for detecting BSJ reads. In CIRI2, the search for potential junction reads could involve multiple redundant scanning steps, especially in large datasets in which repetitive or overlapping read mappings are common. CIRI3 addresses this issue by dividing the alignment file into indexed ‘blocks’ that can be independently searched for junction signatures. Once a junction is identified in a block, related reads are quickly grouped and processed together, dramatically reducing the number of redundant operations. CIRI3 represents a significant advance over CIRI2, offering superior scalability, precision and analytical depth, which make it especially well-suited for large-scale circRNA studies and biomarker discovery.

### CIRCexplorer2

CIRCexplorer2 is another widely used bioinformatics programme designed for the identification and annotation of circRNAs from RNA-seq data [28]. It starts by aligning RNA-seq reads to a reference genome using the splice-aware aligner STAR [63]. Once the reads are processed, CIRCexplorer2 has a parse module that identifies potential BSJs, which reconstructs circRNA candidates by recognising where the 3’ end of a downstream exon is joined to the 5’ end of an upstream exon in a nonlinear manner. The junction reads are then filtered and grouped to define high-confidence circRNAs. The algorithm uses input gene annotation files, such as those from GENCODE [16] or RefSeq [67], to assign circRNAs to host genes, determine their exon composition and define them as exonic, intronic or intergenic. If a gene annotation file is not provided, CIRCexplorer2 can also operate in *de novo* mode. CIRCexplorer2 characterises full-length circRNA isoforms by reconstructing exon structures from BSJ reads and mapping read coverage across the circRNA body. It provides detailed annotations in the output file, including the chromosomal location, strand, host gene and number of supporting junction reads. CIRCexplorer2 can use either the TopHat-Fusion mapping output .bam file [68] or the STAR alignment output StarChimeric.out.junction file [21]. The bias from base offset around exon‒intron junctions can be corrected in the following step. This correction is achieved by embedding a function that uses an annotation file, a reference genome file and a ‘bsj.bed’ file that contains BSJ information [27].

CIRCexplorer2 identifies circRNA-predominant alternative splicing events by comparing paired poly(A) − and poly(A) + RNA-seq datasets from the same source. As circRNAs lack poly(A) tails, poly(A)− data capture the circRNA splicing landscape, whereas poly(A)+ data represent the linear RNA splicing profile. CIRCexplorer2 is also compatible with ribosomal depletion RNA-seq datasets as an alternative to poly(A)− libraries. By calling the ‘—abs’ parameter in CIRCexplorer2, information regarding alternative splicing and 5’ and 3’ backsplicing, including the percent circularised-site usage, can be produced. The combined use of CIRI2 and CIRCexplorer2 is often completed to filter out false positives for their analysis, as described previously [37].

### CIRCexplorer3

CIRCexplorer3 was released in 2020 and builds on CIRCexplorer2 with the addition of a uniform normalisation metric and fragments per billion mapped bases (FPB), which are applied to circRNA and linear RNA data [66]. For circRNAs, reads mapping to the BSJ sites are normalised to the total number of mapped bases. For linear transcripts, reads mapped to the canonical splice junctions are also normalised. This approach is distinct from the CIRCexplorer2 pipeline, in which circRNAs are quantified by fragments per million, while linear RNAs are quantified over exons/splice junction sites. As two different normalisation methods are used, the expression of circRNAs cannot be compared directly with that of their linear counterparts. However, CIRCexplorer3 also computes a CIRCscore, which is a calculated measure of circRNA expression against the expression of its linear counterpart. The CIRCscore is FPBcirc/FPBlinear, with a value ranging from 0 to greater than 1, where a value greater than 1 indicates that the circRNA is expressed at a higher level than its linear counterpart, and a value less than 1 indicates that it is expressed at a lower level than its linear counterpart. While CIRCexplorer2 is still considered the standard tool for circRNA detection, CIRCexplorer3 enables the exploration and comparison of corresponding linear RNA expression profiles.

## Meta-pipelines for circRNA detection

No single caller is universally optimal as individual detection tools each have unique strengths. Meta-pipelines address this by integrating multiple detection tools within a single framework and producing consensus circRNA sets that reduce tool-specific bias and false positives.

In recent years, integrated bioinformatics programmes for circRNA detection have been developed in which a consensus set of circRNAs detected by more than one detection tool has been constructed, limiting the inclusion of false positives in downstream analyses (Table 2). Such integrative programmes include CirComPara2 and circRNAwrap, which integrate tools such as find_circ, CIRCexplorer2 and CIRI2 in one programme [46, 69].

**Table 2:** Meta-pipelines for circRNA quantification

Tool	Scope	Quantification and normalisation	Language
CIRIquant	Quantify circRNA + linear RNA expression; integrates tools	Builds a circRNA pseudoreference; estimates BSJ counts; calculates linear transcript abundance via StringTie; supports RNase-R normalisation, Gaussian mixture model	Python, R
CIRCexplorer3-CLEAR	Unified circRNA vs. linear RNA quantification	Fragments per billion mapped bases (FPB) for circRNAs and linear RNAs; CIRCscore = FPB_circ/FPB_linear	Python
CirComPara2	Consensus detection + quantification	Merges multiple callers; outputs per-tool and consensus BSJ count tables (normalisation/DE left to downstream tools)	Python
CircRNAwrap	End-to-end (FASTQ→circ list)	QC/trim → mapping → caller(s) → merge and abundance	Python
nf-core/circrna (Nextflow)	Best-practice pipeline	Runs multiple callers, merges counts, produces MultiQC; no built-in DE (outputs are DE-ready)	Nextflow (Groovy DSL2), Python, R
CircRNAFlow (Nextflow)	Nextflow pipeline (detect→function)	Detection using DCC/STAR; adds miRNA/RBP/translation modules	Nextflow (Groovy DSL2), Python, R
psirc	Full-length circRNA isoforms	Pseudoalignment and graph-based isoform reconstruction; isoform-level quantification	C++, Python
AQUARIUM	Utilises the outputs of circRNA detection tools to determine the relative abundances of linear RNAs and circRNAs	Transforms circular transcripts into pseudolinear transcripts to estimate the expression of linear and circular transcripts using a salmon framework	Python, R

CircRNAwrap is a meta-pipeline developed to increase the reliability of circRNA detection by integrating multiple existing circRNA identification tools into one unified workflow. The methodology is designed to address the variability and inconsistency observed across individual circRNA detection tools, each of which uses different alignment and filtering strategies. The workflow of circRNAwrap begins with preprocessing RNA-seq data through standard quality control and trimming steps using cutadapt [69]. The multiple circRNA detection tools utilised in circRNAwrap include eight programmes: Acfs, CIRI2, circRNA_finder, DCC, find_circ, CIRCexplorer, MapSplice and KNIFE [28, 65, 70–72]. Most of the tools use common alignment tools, such as BWA, STAR, Bowtie2 and samtools [57, 61, 63, 73]. CIRI, RAISE, CIRCexplorer2 and Sailfish-cir are used for sequence prediction and the assessment of the abundance of circRNAs [28, 60, 74, 75]. With respect to the methodology, the outputs of four different aligners were subsequently input into eight different circRNA detection tools. Three different tools for circRNA transcript prediction are used, followed by abundance estimation with Sailfish-cir. The main advantage of circRNAwrap lies in its consensus-based approach, which significantly improves the robustness of circRNA detection by leveraging the complementary strengths of different algorithms. It is particularly useful for reducing tool-specific biases and identifying high-confidence circRNAs that are consistently supported across multiple methods. It offers a flexible, modular and reproducible framework that allows more reliable circRNA discovery and comparisons across samples and studies. Overall, the developers of circRNAwrap found that the outcomes of circRNA analyses are highly dependent on the computational tools and alignment strategies applied, and no single method has proven universally superior for reliable circRNA identification and characterisation.

### CirComPara2

CirComPara2, which was developed in 2021, expands on circRNAwrap and has the distinction of including quality control and trimming of reads, as well as a tangible linear RNA analysis pipeline [29]. CirComPara2 combines 15 bioinformatics programmes across the categories of quality control, alignment and circRNA detection. CirComPara2 utilises FastQC for quality assessment and several tools for alignment, such as HISAT2, STAR, BWA, Bowtie/2, TopHat and Segemehl [57, 61, 63, 68, 76, 77]. For circRNA detection, the programme incorporates CIRI2, CIRCexplorer2, find_circ, circRNA_finder and DCC [26–28, 58, 71]. By combining multiple detection tools, CirComPara2 leverages their complementary strengths to improve detection, sensitivity and reliability. Each combination of aligner and caller targets different properties of circRNA reads, such as the BSJ, read orientation, anchor length and mismatch tolerance, thus capturing a more comprehensive circRNA landscape. In CirComPara2, CIRCexplorer2 uses annotated junctions and TopHat-Fusion for detecting backsplicing events, which rely on annotation and spliced sequence alignment. CIRI2 uses BWA-MEM and a maximum entropy model to detect circRNAs. DCC builds on the ability of the STAR aligner to identify chimeric alignments and applies stringent filtering criteria based on mapping quality. find_circ scans for anchor reads split across a BSJ using Bowtie and filters out false positives based on empirical rules. Segemehl, a nonsplice-aware aligner, uses a suffix array to align reads that map discontinuously or in a scrambled order and is a highly sensitive tool for circRNA detection, particularly in non-annotated regions.

The use of multiple circRNA detection tools enables the creation of a more dependable circRNA list that can be used for downstream analysis, such as differential expression and functional annotation. Both the circRNA and its linear counterpart can be used for differential expression and Gene Ontology analyses and can facilitate a further exploration of the roles of circRNAs in biological contexts. CirComPara2 controls the number of false positives by considering only the circRNAs commonly detected by two or more methods. The developers of CirComPara2 demonstrated that an average of 49% of false negatives detected using each method showed higher expression than the overall median circRNA expression, suggesting that nearly half of the missed circRNAs had a high expression level, regardless of the method applied. The expression profile of the false negatives closely resembled that of the correctly detected circRNAs, representing true positives, while the expression levels of false positives were typically low. An examination of the circRNAs that were undetected by one or more tools revealed that only 4% of the circRNAs within the false-negative group were missed by all the methods, while the remaining 96% were detected by at least one of the nine tools.

Compared with the methods using single tools, the combined approach of CirComPara2 produces more comprehensive and more stable predictions. Even after stringent low-count filters are applied, CirComPara2 continues to recover circRNAs that would otherwise be overlooked, indicating that these additionally detected circRNAs are not simply low-abundance artefacts but are biologically relevant transcripts.

### Nextflow nf-core/circRNA

The Nextflow nf-core/circRNA detection pipeline designed for a comprehensive analysis is composed of three core analytical modules: circRNA discovery and quantification, miRNA target prediction and differential expression analysis [78]. The methodology works by inputting RNA-seq reads that undergo quality assessment with FastQC and adapter/quality trimming using Trim Galore [79]. For the detection of circRNAs, multiple tools, including circRNA_finder, CIRCexplorer2, DCC, find_circ, MapSplice and Segemehl, are integrated to detect BSJs following the STAR two-pass alignment optimised for chimeric junction identification [27, 28, 58, 71, 72, 77]. Candidate circRNAs are filtered based on minimum read support, prevalence and cross-caller concordance and then merged to generate one unified BSJ set. An annotated GTF file is used, with circular transcript sequences reconstructed to construct a circular transcriptome, followed by quantification using the linear reference transcriptome. The expression levels of both circular and linear transcripts are measured using psirc and/or CIRIquant, with a bootstrap-based estimation applied when relevant [29]. For the downstream regulatory analysis, miRNA binding sites are predicted using miRanda and TargetScan, with the optional integration of external miRNA expression data to compute circRNA‒miRNA correlations [80, 81]. CircRNA expression is evaluated using CircTest [58]. All outputs, such as circRNA detection results, annotations, quantifications and predicted miRNA interactions, are compiled and summarised in an integrated report using MultiQC. For miRNA predictions, target miRNAs must be called by both the target prediction algorithms miRanda and TargetScan to reduce the number of spurious calls. Notably, DCC produced a high proportion of circRNAs that were not covered by other tools, suggesting a high rate of false positives, which was confirmed with a 75.55% precision score [78]. The application of sensible parameters and supplementation with other quantification tools are recommended to reduce the number of false positives. The Nextflow nf-core/circRNA pipeline is among the few tools used to analyse circRNAs that incorporate a differential expression analysis within the same framework. For each comparison, the pipeline identifies both up- and downregulated circRNAs and provides comprehensive visual outputs, including heatmaps, volcano plots and MA plots, and distributions of raw and adjusted p values. An MA plot is a visualisation tool that uses results from differential expression to plot M (log fold change between two conditions) and A (average mean expression across samples). In addition, for every differentially expressed circRNA detected, boxplots are generated to illustrate normalised expression across the experimental groups. The workflow simultaneously performs a differential expression analysis of linear mRNA transcripts to complement these results. Interestingly, the researchers assessed quantification tools that negatively affected the performance in each set and found that DCC negatively affected the performance metrics, which were tested using a t-test, and a negative correlation was observed between the loads for precision and the F1 score [78]. DCC is the only quantification tool that utilises the STAR 2-pass mode, greatly increasing the sensitivity around splice junction sites at the cost of false positives [82]. Nextflow nf-core/circRNA is a portable workflow that quantifies circRNAs, performs miRNA target predictions and analyses the differential expression of circRNAs in a single execution.

## Cross-benchmarking circRNA detection tools

The diversity of available detection tools and meta-pipelines raises the practical question of which tools are optimal for defined conditions. Recent large-scale benchmarking efforts have directly addressed this question and have informed tool selection across the pipeline. Vromman et al.’s study (2023) benchmarked 16 circRNA detection tools against each other, including CIRCexplorer3 [66], CirComPara2 [46], circRNA_finder [46], circseq_cup [83], CircSplice [84], circtools [85], CIRI2 [26], CIRIquant [29], ecircscreen [22], find_circ [27], KNIFE [65], NCLscan [54], NCLcomparator [86], PFv2 [87], Sailfish-cir [75] and segemehl [77]. More than 315,000 unique circRNAs were predicted by the listed tools, of which they were ran on three human cancer cell lines and validated with reverse transcription quantitative PCR (RT-qPCR), RNase-R treatment and amplicon sequencing. A 40-fold difference was observed between the tool with the highest prediction of circRNAs (circseq_cup with 58,032 circRNAs detected) and the tool with the lowest prediction (segemehl with 1,372 circRNAs detected) for one of the cell lines. The researchers also found that half of the total circRNAs detected in the study (49.9%) were reported by only one tool. Analysing the tools individually, circseq_cup, KNIFE, NCLscan and NCLcomparator reported greater numbers of novel circRNAs (87.8%, 53.9%, 53.4% and 53.3%, respectively). CIRI2 had the greatest number of circRNAs detected, with a BSJ count above and below five and with approximately 55,000 circRNAs detected. CIRIquant, CircSplice and find_circ attained approximately 42,000 circRNAs detected, with similar results reported for BSJ above and below five. CirComPara2, CIRCexplorer3 and circtools detected fewer circRNAs, approximately 25,000. This was followed by NCLscan, NCLcomparator, PFv2 and ecircscreen, with approximately 18,000 circRNAs detected. KNIFE, circRNA_finder and segemehl had the lowest number of detected circRNAs, reaching less than 10,000. The researchers noted that higher precision proceeded from their presence of the circRNAs detected in multiple tools. However, the often-used practice of using at least two tools is not necessarily a guarantee of avoiding false-positive results. Tool-specific precision was consistently high across all methods, with median values of 98.8% (qPCR), 96.3% (RNase-R) and 95.5% (amplicon sequencing), suggesting high precision. Sensitivity was the primary differentiator between the tools. These findings have direct implications for downstream analyses of coding potential, as any circRNA missed at the detection stage cannot be recovered by subsequent prediction tools such as CPC2, CPAT or CICADA, regardless of their accuracy. The benchmarking paper underscores the importance of tool selection at each stage of the analytical pipeline and suggest that combining complementary detection tools may provide a more comprehensive set of candidate circRNAs for the coding potential evaluation.

## Annotation and visualisation of circRNAs

Once a confident circRNA set has been assembled, downstream interpretation requires tools that annotate and visualise the detected circRNAs’ structural and regulatory features. This category of tools bridges raw detection output and functional analysis by providing information regarding exonic structure, regulatory element context, primer design support and graphical representations of BSJs.

### circPrimer2.0

The Java-based software tool circPrimer2.0 was designed to annotate circRNAs and predict their translation potential. Its key features include the annotation and visualisation of circRNAs using the circBase ID, genomic coordinates or gene symbols. It retrieves the spliced exonic sequence and produces images of the structure of circRNAs with exon/intron boundaries and BSJs [88]. The software also supports the design of divergent primers, including one that spans the BSJ. The ‘check primer’ module can also evaluate designed primer sequences, report which circRNAs are likely to be amplified by those specific primers and show their binding location relative to the circRNA structure. For each circRNA sequence, circPrimer2.0 scans all frames for the start and stop codons, including ORFs that span the BSJ. It also handles infinite ORFs when the circRNA length is divisible by three or, in other cases, with a frame shift. circPrimer2.0 predicts whether fragments of circRNA likely contain an active IRES using a machine learning model built with XGBoost trained on known native IRES sequences with global k-mer features as input. If multiple candidate fragments are identified, the one closest to the predicted start codon is used. XGBoost is a model that enables the highly accurate and efficient classification of coding potential and circRNA translational likelihood [89]. The authors observed that IRES elements embedded within long nucleotide sequences often yielded false-negative predictions due to the dilution of the k-mer feature signals caused by the surrounding non-IRES regions. circPrimer2.0 splits the full circRNA sequence into fragments of 174 nt with a step size of 20 nt to address this issue, resulting in adjacent fragments sharing a 154 nt overlap [88]. The 174 nt unit was selected due to Gritsenko et al.’s study which suggests the length of sequence was limited to 174 nt, which is shorter than some reported long structural IRESs [90]. K-mer frequencies are then calculated for each fragment independently. If two or more fragments are predicted to contain IRES elements, the IRES located nearest to the start codon is designated as the putative translational initiation site for the corresponding ORF. Notably, a positive prediction indicates that the fragment contains an IRES rather than the entire 174 nt region functioning as an IRES. circPrimer2.0 also incorporates known m6A modification sites, which have been implicated in the cap-independent translation of circRNAs. Rather than predicting m6A sites computationally, the software retrieves experimentally validated sites from the m6A-Atlas database and displays all DRACH/RRACH consensus motifs within a given circRNA sequence. This approach allows users to assess whether a candidate ORF is located near a potential m6A-driven translation initiation site. One of the benefits of circPrimer2.0 is that it has a graphical user interface and command-line interface, producing graphs and schematics of circRNAs and their ORFs and IRESs. As circPrimer2.0 provides peptide sequences for each ORF in a circRNA, the output can be supplied to CPAT and CPC2 to determine the protein-coding potential. The circRNA fasta file generated by circPrimer2.0 can be used in linear protein coding tools, such as CPC2 and CPAT, along with their chromosome coordinates, to determine the protein coding potential.

### Visualisation tools for circRNAs

Visualisation tools have become an essential component of circRNA research, giving investigators the means to interpret detection outputs in their broader biological context and to prioritise candidates for functional follow-up. Several bioinformatics tools have been developed to visualise circRNAs and explore their structural and functional features, each of which caters to different aspects of circRNA biology. CircView is among the user-friendly tools developed for exploring circRNAs detected by multiple pipelines, enabling users to inpsect individual circRNAs alongside regulatory features, such as miRNA response elements and RNA-binding protein (RBP) binding sites [91]. Ularcirc extends this by integrating the visualisation of both back-splice and forward splice junctions, allowing users to compare circRNA expression against its parental linear transcript and explore internal exon usage and ORFs [92]. It implements the output of CIRI, CIRCexplorer or raw chimeric output of the STAR aligner and assembles the BSJ count table to allow multi-sample analysis. circtools provides a broader one-stop framework with modules for quality control, primer design, exon usage and reconstitution of full-length circRNAs, making it particularly useful for a downstream experimental design [85]. Rcirc, implemented as an R package, focuses on the coding potential of circRNAs and offers both single-circRNA and meta-feature visualisations across thousands of candidates [93]. It provides many highly automated functions without requiring the user to perform many complicated processes. Recently, CircNetVis has addressed the growing interest in circRNA regulatory networks by providing an interactive web application interface for visualising circRNA–miRNA, miRNA–mRNA and circRNA–RBP interactions [94]. On the other hand, CircPac offers a web-based platform that integrates data from multiple circRNA databases for streamlined exploration and visualisation [95]. Complementing these standalone tools, CIRCpedia v3 functions as both a curated database and an interactive visualisation platform, enabling cross-species comparisons, expression profiling against cognate linear transcripts and integrated functional predictions [96].

## Protein-coding potential of circRNAs

While the tools discussed so far identify, contextualise and visualise circRNAs, an equally important analytical layer concerns whether these transcripts have protein-coding potential. This section describes the tools developed for assessing the translatability of circRNAs, from general-purpose coding potential calculators to recent circRNA-specific machine learning frameworks.

The protein-coding potential of circRNAs has been consistently documented in numerous studies, expanding current functionalities in cellular processes [4]. The characterisation of circRNAs as functional molecules capable of protein synthesis has shifted their perception from being splicing artefacts to vital components of gene expression regulation. Recent research has shown that certain circRNAs harbour ORFs that can potentially direct the translation of functional peptides [97]. Other studies have highlighted the translational ability of circRNAs under specific cellular conditions [98]. Typically, ORFs are transcribed and subsequently translated to produce annotated proteins with predictable functions. However, research has demonstrated that a subset of ORFs lacks protein-coding capacity in the conventional sense, instead giving rise to small peptides upon translation [99]. Another way that circRNAs can initiate translation is through an IRES that directly recruits the ribosome internally, bypassing the need for the 5’ cap [100]. When a circRNA contains an IRES, it can bind to a small ribosomal subunit and associated initiation factors to form a functional translation initiation complex. This complex enables ribosomes to recognise an internal AUG start codon and initiate protein synthesis from within the circRNA [101]. CircRNAs, particularly *circZNF609* and *circFBXW7*, have been validated as protein-coding circRNAs whose cap-independent translation is downregulated, making IRES-mediated translation a key alternative pathway for protein production [97, 102]. Programmes designed for the detection of the protein-coding potential of circRNAs are summarised in Table 3 and discussed in detail in the following section.

**Table 3:** Tools for predicting the protein-coding function of circRNAs

Tool	Core model/features	Coding potential output	Language
CPC2	SVM on four intrinsic features (Fickett score, ORF metrics, and hexamer usage)	Binary coding/noncoding score	Python
CPAT	Logistic regression using ORF size/coverage, Fickett score, hexamer bias	Probability of coding potential	Python
CircProPlus	Integrates circRNA calls with Ribo-seq/MS; ships updated resources; default detection via CIRI2	Ranked translatable circRNA candidates	Python
CircCode3	Detects BSJ-spanning ORFs; evaluates IRES (IRESfinder), m^6^A (DeepCircM6A), stop codon reliability (DLMSC); ML integration	ORFs with integrated evidence and visuals	Python
CICADA	ML detects HPCRs and constructs ANTfeatures; predicts with random forest models; outputs peptide candidates	Coding potential scores + peptide libraries	Python

Abbreviations: SVM – support vector machine, ORF – open reading frame, MS – mass spectrometry, BSJ – back-splice junction, IRES – internal ribosome entry sites, ML – machine learning, m^6^A – N6-methyladenosine, HPCR – high-potential coding regions, ANT – adjoining nucleotide triplet

### Coding potential calculator 2

Coding Potential Calculator 2 (CPC2) is a computational tool designed to assess the protein-coding potential of linear RNA transcripts using intrinsic sequence features [103]. CPC1, which was originally published in 2007, relies on sequence alignment to known protein databases, while CPC2 employs an alignment-free approach, with higher speed and applicability across different species, including those whose genomes are poorly annotated. Four key sequence features are used to calculate the protein-coding potential score: ORF length, ORF integrity, Fickett score and isoelectric point (pI). CPC2 identifies the longest ORF within a transcript to serve as its primary feature. ORF integrity involves assessing whether the ORF possesses both a canonical start codon (AUG) and an in-frame stop codon (UAA, UAG or UGA) to determine a complete and potentially functional coding score [104]. The Fickett score is derived from the nucleotide composition and positional nucleotide frequencies and is used to evaluate coding potential based on patterns commonly observed in protein-coding regions. CPC2 translates the longest ORF into a hypothetical peptide and then calculates the pI from that amino acid sequence, providing insights into the physiochemical properties of the predicted peptide, which can differ between coding and noncoding sequences. The model was trained on the four intrinsic features with the LIBSVM package using the standard radial basis function kernel with 17,984 high-confidence human protein-coding transcripts and 10,452 noncoding transcripts. Support vector machines (SVMs) are a set of supervised learning methods used for classification, regression and outlier detection. The LIBSVM is an open-source library for support vector classification and regression that implements a sequential minimal optimisation-type decomposition method with support for multiple kernel functions, such as linear, polynomial, radial basis function and sigmoid. It can perform cross-validation, probability estimation and multiclass classification [105]. It produces probability outputs that CPC2 uses to calculate a coding probability score rather than a binary call. While CPC2 was constructed for linear transcripts, its alignment-free methodology, which is based on intrinsic sequence features, makes it suitable for evaluating the protein-coding potential of the circRNAs.

The chromosome coordinates of differentially expressed sequences can be used as an input in circPrimer2.0, which generates a circRNA fasta file that can be employed as an input for CPC2 [88]. It is evaluated using the four key parts to distinguish coding from noncoding circRNAs. CPC2 is a highly accurate protein-coding detection tool for all transcripts in general that have been found to be key regulators of several physiological and pathological processes. Overall, CPC2 operates independently of external databases by utilising four intrinsic sequence features that are interpretable and biologically relevant. At the DNA level, the Fickett score quantifies positional nucleotide preferences within the sequence. At the RNA level, ORF length and ORF integrity serve as strong indicators of coding potential, as genuine protein-coding transcripts typically contain long, intact ORFs [106]. Based on the assumption that peptides translated from noncoding transcripts exhibit distinct biochemical properties compared with those encoded by authentic protein-coding genes, additional peptide-level descriptors were initially evaluated, and the pI of the hypothetical peptide was incorporated as the fourth feature in the final SVM model to improve classification performance.

### Coding-potential assessment tool

The Coding-Potential Assessment Tool (CPAT) is another alignment-free bioinformatics tool designed to differentiate protein-coding transcripts from ncRNAs using intrinsic sequence features. CPAT was developed by Wang et al. and is used for classifying transcripts, particularly in large-scale transcriptome studies in which traditional alignment-based methods may be computationally intensive or less effective for novel sequences [107]. It uses a logistic regression model trained on known coding and noncoding sequences using four parts similar to CPC2: ORF size, ORF coverage, Fickett score and hexamer usage bias. While the ORF size and Fickett score are similar to those of CPC2, CPAT uses ORF coverage and hexamer usage bias in place of the CPC2 ORF integrity and pI. The Fickett TESTCODE statistic score evaluates the compositional bias and positional nucleotide frequencies within a sequence, capturing patterns characteristic of coding regions. It is obtained by computing four position values and four composition values (nucleotide content) from the DNA sequence. The position value reflects the degree to which each base is favoured in one codon position over another. The percentage of each nucleotide within the sequence is also calculated as four composition values. Eight values are converted into probabilities of coding using a lookup table, in which each probability is then multiplied by a weight (*w*). The value of *w* reflects the percentage of time each parameter alone successfully predicts the coding or noncoding function for sequences with known functions. Unlike ORF-based features, the Fickett score is derived from the full-length transcript sequence, providing an independent measure of coding potential. When the test region is more than 200 nucleotides in length, which includes the longest ncRNAs, the Fickett score alone can achieve 94% sensitivity and 97% specificity. The hexamer usage bias or hexamer score may be the most discriminating feature because of its dependence on adjacent amino acids in proteins. It is calculated as a log-likelihood ratio that measures differential hexamer usage between coding and noncoding sequences.

The same approach of generating a circRNA fasta file from circPrimer2.0 is needed for use as an input for CPAT to be applied to circRNAs. CPAT then identifies the longest ORF within the reconstructed sequence, calculates the ORF coverage, calculates the Fickett score and analyses the usage of hexamer motifs that differ between coding RNAs and ncRNAs. While CPAT does not directly account for circRNAs, it uses circRNA gene coordinates and a circRNA fasta sequence that can be evaluated. Overall, the classification performance of CPAT is superior to that of the other evaluated tools, namely, CPC1, PhyloCSF and the protein-independent SVM model (PORTRAIT) [108]. Although CPC1 achieved the highest sensitivity (0.99), its specificity was relatively low (0.74) [109]. Thus, while CPC1 was highly effective in correctly identifying protein-coding transcripts, it frequently misclassified ncRNAs as coding, as approximately 26% of true noncoding transcripts were incorrectly assigned coding potential. One possible explanation is that CPC1 relies on sequence alignment to known protein databases, and a considerable fraction of ncRNAs overlap with either the sense or antisense strand of protein-coding genes, producing significant alignment hits that lead to false-positive coding calls. By contrast, the alignment-free approach of CPAT prevents this source of misclassification, achieving a more balanced sensitivity (0.96) and specificity (0.97). Compared with existing methods, CPAT offers a higher computational speed and greater ease of use. CPAT can efficiently and accurately evaluate the coding potential of tens of thousands of transcripts in real time, making it a valuable resource for the expanding RNA-seq research community.

### CircProPlus

CircProPlus is an advanced bioinformatics programme that identifies and characterises protein-coding circRNAs [110]. Its modular architecture allows for flexibility and customisation across several bioinformatic stages, each relying on specialised programmes. The programme provides users with the choice of multiple circRNA detection tools and employs both CPC2 and CPAT to calculate protein-coding potential robustly. It is an automated computational pipeline that integrates circRNA detection, coding potential prediction using CPC2 and CPAT and the identification of ribosome-protected fragments through the alignment of Ribo-seq reads to circRNA sequences. By combining a sequence-based assessment of coding potential with translational evidence from Ribo-seq data, CircProPlus provides a comprehensive evaluation of the protein-coding potential of circRNAs compared with any single tool. Three modules comprise CircProPlus: circRNA identification (module one), coding potential score calculation (module two) and Ribo-seq read identification (module three). In module one, CIRI2 (default) or CirComPara2 is used to detect circRNAs. As previously discussed, CirComPara2 consists of multiple circRNA detection tools, including CIRCexplorer2, find_circ, DCC and CIRI2, to provide a consensus list of nonredundant circRNAs supported by at least two methods. The coding potential score is then calculated for identified circRNAs using both CPC2 and CPAT for cross-validation. Finally, CircProPlus is used to analyse the Ribo-seq data to provide experimental evidence of translation. This module incorporates the supplied Ribo-seq reads and previously calculated coding potential scores to identify protein-coding circRNAs. The methodology aligns Ribo-seq reads to circRNA sequences to confirm ribosome engagement, thus validating the translational potential of the circRNAs. Here, the Ribo-seq reads can align to any part of the circRNA other than the BSJ. Overall, CircProPlus provides an automated framework for the detection of circRNAs and their protein-coding potential. By integrating an analysis of structural features and experimental validation using RNA-seq and Ribo-seq data, the reliability of circRNA protein-coding potential can be enhanced. Its scalability and efficiency make it suitable for large-scale transcriptomic analysis.

### CircCode3

Developed in 2025, CircCode3 detects the protein-coding potential of circRNAs by integrating ribosome profiling and machine learning techniques. CircCode3 is the latest version of CircCode, which was originally released in 2019 [30, 111]. In the latest version of CircCode3, the identification and assessment of ORFs spanning BSJ sites were introduced. IRESfinder was also added to the bioinformatic pipeline [109]. It works by preprocessing Ribo-seq reads, which include adaptor trimming, quality control, removal of ribosomal RNA contaminants and alignment using algorithms such as Bowtie2. The cleaned reads that map across the BSJs are retained as data for potential translation events, while candidate circRNAs are cross-referenced with mass spectrometry peptide data to increase reliability. CircCode3 identifies Ribo-seq read-mapped regions on junctions and predicts ORFs spanning BSJs. Each candidate ORF is further characterised using multiple tools: IRESfinder, CPC2, CPAT and ORFfinder [103, 107, 109, 112].

Zhu et al. also introduced and developed two deep learning tools: DeepCircm6A for the prediction of m^6^A modification sites in circRNAs and Deep Learning Model of the Stop Codon (DLMSC) for assessing the reliability of stop codons. The output file contains detailed information, including aligned reads or detected peptides, m^6^A modification sites, ORF locations, translation details and evaluation outcomes. Users can also generate visual representations of these ORFs for further analysis. DeepCircm6A uses a deep learning architecture that combines convolutional neural networks and bidirectional long short-term memory layers to predict m^6^A modification sites within circRNAs, achieving an AUC of 0.99 with cross-species applicability. DLMSC evaluates the reliability of stop codons within circRNA ORFs and achieves 90.1% accuracy on an independent mouse test dataset from RiboCirc, demonstrating cross-species effectiveness. When CircCode3 is compared with CircCode on simulated data, CircCode3 outperforms CircCode across all the metrics except recall, achieving 99.73% accuracy, 99.68% precision and 99.73% F1 score, respectively, compared with the 96.55% accuracy and 93.55% precision of CircCode [30]. CircCode3 also generates graphical visualisations, such as linear and base-level circRNA maps that display read coverage, predicted ORFs, IRES elements and modification sites. By combining Ribo-seq and mass spectrometry tools with advanced feature extraction and deep learning models, CircCode3 provides a systematic platform for the characterisation of translatable circRNAs.

### CircRNA-coding ability and product detection algorithm

The circRNA-coding ability and product detection algorithm (CICADA) is a machine learning model designed to distinguish protein-coding circRNAs from noncoding circRNAs based on sequence-intrinsic features while accounting for circRNA specificity [113]. Unlike CPC2 and CPAT, which were developed for linear transcripts, CICADA incorporates circRNA-specific features, including IRES elements and m^6^A modification sites, which underlie cap-independent translation initiation in circRNAs [113]. The highest potential coding region (HPCR) of each circRNA is subsequently used to identify potential peptide products (circProts), with ORFs classified based on the presence or absence of start and stop codons. Translatable circRNAs can be categorised into three types based on the location of their coding regions and the mechanism of translation: those undergoing rolling-circle translation, those translated across the back splice junction and those translated across the back splice junction without rolling-circle translation. CICADA concatenates each circRNA sequence multiple times to model rolling-circle translation, a hallmark of circRNA-derived peptide production. A conservation feature is also used in training the model to determine how well the HPCR sequence of a circRNA is evolutionarily conserved across species. The mean conservation score is calculated using phyloP and/or phastCons [114, 115]. The inputs for CICADA require full-length circRNA sequences and yield binary results with or without coding potential. CICADA has a key practical advantage over tools such as CircProPlus and CircCode3, which require Ribo-seq data that are expensive and technically demanding to generate. When benchmarked against CPC2, CPAT and the coding–noncoding index on a test set of 193 experimentally verified translatable circRNAs, CICADA achieved the highest accuracy, with an area under the receiver operating characteristic curve of 0.951 and an area under the precision‒recall curve of 0.865, outperforming all three tools in terms of the F1 score, geometric mean and the Matthews correlation coefficient [116]. Compared with Ribo-seq-dependent pipelines, such as CircPro and CircCode, CICADA produced a comparable number of circRNA-encoded products supported by mass spectrometry evidence, demonstrating that a sequence-based prediction alone can achieve results similar to those of more resource-intensive approaches.

## Limitations

Although new bioinformatics tools for circRNA quantification and detection are being developed more frequently, their approaches still have limitations. At the data preprocessing stage, performing quality checks on datasets before they are used in circRNA tools is always critical. Mismatched chromosome naming and annotations can generate falsely detected circRNAs. Misaligned short reads can also introduce errors that propagate throughout the downstream analysis. At the circRNA detection stage, CIRI2/CIRI3, CIRCexplorer2, DCC and find_circ depend on the properties of their alignment tools, BSJ filters and parameters that change the sensitivity and specificity across datasets [26–28, 49, 58]. BSJs can be missed when the anchor lengths are too short or when the mapping parameters are too strict. DCC uses STAR, which, if configured leniently, can falsely increase the number of BSJs; inversely, if the parameters are too tight, it can decrease sensitivity [58]. While find_circ is a classic and fast approach, its simplicity can result in higher false positives in repetitive regions and low read counts [27]. Meta-pipeline detection tools, such as CirComPara2, reduce false positives by employing multiple tools and aligners. However, these tools are subject to different parameters and annotations that can vary across datasets. At the coding potential prediction stage, back splice-spanning ORFs in circRNAs can be misclassified as noncoding. CICADA addresses this issue by incorporating circRNA-specific features, but its random forest model is trained using intronic sequences as the negative dataset rather than noncoding circRNAs [113]. As intronic sequences are compositionally distinct from exon-derived circRNAs, the model may struggle to discriminate between coding and noncoding circRNAs in practice, as evidenced by independent benchmarking, in which the accuracy of CICADA decreased to 73.22% when it was tested against exon-derived negative sequences [116]. Sliding window detection can also oversegment repetitive regions, increasing the number of false positives. CircProPlus and CircCode3 require Ribo-seq reads and RNA-seq reads to detect circRNA translation, which can be difficult to obtain [30, 110]. Given these limitations across each stage, a cross-modal workflow in which bioinformatics outputs are verified by targeted molecular assays would be advantageous for confirming circRNA translation.

Detection accuracy remains highly dependent on sequencing depth, library preparation and alignment strategy. CircRNA detection methods that utilise short-read RNA-seq data have various drawbacks, such as overlooking short ORFs that may contain BSJs and difficulty distinguishing circRNA isoforms derived from the same BSJ [117]. In addition, as many circRNAs are multiexonic, the internal sequence and exon composition remain undetermined [118]. However, the use of full-length circRNAs with long-read sequencing technology can overcome such limitations by enabling the characterisation of the internal composition and splicing patterns of circRNAs. Differences in read mappability and reference annotation can also lead to inconsistent circRNA identification across tools and studies, limiting reproducibility. Quantification and isoform reconstruction remain challenging for researchers. Many circRNAs arise from complex splicing events that generate multiple isoforms that share the same back splice junction. Current pipelines typically collapse these isoforms into a single entity, obscuring isoform-specific expression and functional diversity. In addition, normalisation and expression estimation are complicated by the absence of poly(A) tails and variable read coverage across junctions, which affects downstream differential expression and coexpression analyses. In the context of the prediction of translational potential, tools such as CPC2 and CPAT rely on handcrafted sequence features derived from linear transcripts. These models often fail to distinguish between noncanonical translation events and true coding circRNA**s**, particularly those utilising IRESs or m^6^A-mediated cap-independent initiation. While CircCode3 additionally incorporates mass spectrometry data to provide peptide-level evidence, its reliance on BSJ-spanning peptides constrains its ability to detect circRNAs that lack sufficient proteomic coverage.

## Conclusions

The identification of the protein-coding potential of circRNAs is still an emerging area in molecular biology, with new bioinformatics tools being developed frequently to address the challenges of circRNA detection, quantification and translational assessment. At the detection stage, alignment-based tools such as CIRI2 and CIRCexplorer2 identify circRNAs from RNA-seq data, while integrative frameworks such as CirComPara2 and CircProPlus combine multiple algorithms to improve sensitivity and reduce false positives. At the coding potential prediction stage, sequence-based classifiers such as CPC2 and CPAT provide rapid assessments of translational likelihood, while the more recent circRNA-aware tools, including CICADA, CircCode3 and circPrimer2.0, incorporate features specific to circRNA biology. Each tool differs in its algorithmic approach, and no single tool currently addresses all aspects of circRNA translation prediction. As demonstrated by independent benchmarking studies, the tool choice at every stage of the pipeline significantly influences the downstream results, and the use of complementary tools in combination remains the most reliable strategy for minimising false positives and maximising detection sensitivity.

The future of detecting the protein-coding potential of circRNAs could be improved with the development and advancement of machine learning and deep learning models trained on experimentally validated datasets to provide a better representation of circRNA-specific features. The rapid evolution of computational tools for circRNA detection and the prediction of their translational potential have substantially advanced our understanding of circRNA biology. Coding potential assessment tools, such as CPC2 and CPAT, apply sequence-derived features and statistical models to distinguish coding from noncoding transcripts, with advanced pipelines such as CircCode3 and CircProPlus integrating Ribo-seq evidence to identify actively translated circRNAs. In the future, artificial intelligence and machine learning are poised to revolutionise circRNA research by learning complex, high-dimensional patterns that traditional models cannot capture. These approaches could enable the specific identification of translatable circRNAs, reveal hidden regulatory signals such as IRESs and m^6^A-driven translation and elucidate context-specific translation mechanisms across tissues and disease states.

Emerging single-cell and spatial transcriptomic methods have the potential to map circRNA expression patterns at a cellular resolution, but the low abundance of circRNAs in these contexts remains a technical barrier. The integration of AI-powered multiomics algorithms represents a promising direction, as these approaches could connect the circRNA protein-coding potential to gene activity, epigenetic modifications and regulatory function across tissues and disease states. Currently, deep learning models are designed for linear RNAs and are not directly applicable to circRNAs, as they do not account for circularisation constraints, BSJ structures or the distinct biogenesis and regulatory mechanisms unique to circular transcripts. Therefore, the development of circRNA-specific AI models trained on the growing volume of experimentally validated circRNA datasets will be essential for advancing the characterisation and functional prediction of translatable circRNAs. Advancing the identification of protein-coding circRNAs will depend on the development of computational tools built specifically for circular transcript biology, trained on growing experimentally validated datasets and integrated with multi-omics data. As these approaches improve, our understanding of circRNA function in both normal physiology and disease will also improve, with direct implications for biomarker and therapeutic research.

## Data Availability

No datasets were generated or analysed during the current study.
